# Coaching Leadership and Employees’ Bootlegging Innovation Behavior in Chinese High-Tech Enterprises

**DOI:** 10.3390/bs16040484

**Published:** 2026-03-25

**Authors:** Yueying Wang, Myeong Cheol Choi, Won Gyu Lee, Hann Earl Kim

**Affiliations:** 1 School of Business, Xijing University, Xi’an 710123, China; wangyueying012@163.com; 2 College of Business, Gachon University, Seongnam 13120, Republic of Korea; hk3624@gachon.ac.kr; 3 Department of Intellectual Property Convergence, Gyeongsang National University, Jinju 52828, Republic of Korea

**Keywords:** coaching leadership, bootlegging innovation behavior, work meaning, organizational psychological ownership

## Abstract

This study investigates the psychological mechanisms through which coaching leadership influences employees’ bootlegging innovation behavior. Drawing on leadership and organizational behavior theories, we propose a serial mediation model in which work meaning and organizational psychological ownership jointly transmit the effects of coaching leadership on employees’ informal innovative behavior. Using survey data collected from 427 employees, the results demonstrate that coaching leadership positively predicts bootlegging innovation behavior. Moreover, both work meaning and organizational psychological ownership independently mediate this relationship. Importantly, the findings further support a sequential mediation pathway, indicating that coaching leadership enhances employees’ perceptions of work meaning, which subsequently fosters stronger organizational psychological ownership and, in turn, stimulates bootlegging innovation behavior. By elucidating the intertwined motivational and ownership-based psychological processes underlying informal innovation, this study advances the literature on coaching leadership and employee-driven innovation. The findings also offer practical insights for managers seeking to cultivate grassroots innovation by fostering meaningful work experiences and a sense of psychological ownership among employees.

## 1. Introduction

In the contemporary knowledge economy, in which rapid technological advancements and dynamic market demands continuously reshape competitive landscapes, innovation is an indispensable engine for ensuring organizational sustainability, adaptability, and long-term competitive advantage (Almrshed et al., 2023). Continuous innovation is now an important characteristic of high-performing firms, as they become increasingly concerned with maintaining strategic flexibility and resilience in uncertain environments (Weick & Sutcliffe, 2011; Zighan & Ruel, 2023). While formal innovation processes, such as structured research and development initiatives and top-down innovation management systems, remain essential for coordinating large-scale innovation efforts, a growing stream of organizational behavior research underscores the complementary and often critical role of informal, self-initiated innovation behavior by employees beyond the formal boundaries of their assigned roles (Nanyangwe et al., 2021; Valtonen et al., 2023). One particularly salient form of informal innovation behavior is bootlegging, a discretionary, proactive, and unapproved innovation activity undertaken by employees, typically without the explicit knowledge or sanctions of organizational authority. Bootlegging innovation behavior, as conceptualized by Globocnik and Salomo (2015), reflects employees’ autonomous pursuit of novel ideas, solutions, or products when formal approval may be absent or managerial responses are ambiguous. Despite its covert nature, bootlegging is not inherently subversive; rather, it represents a constructive and entrepreneurial form of employee-driven innovation that enables organizations to benefit from bottom-up creativity, particularly in contexts marked by environmental uncertainty, structural inertia, and bureaucratic rigidity. Extant research demonstrated that bootlegging innovation behavior contributes meaningfully to organizational learning, resilience, and dynamic capability development by circumventing formal constraints and enabling employees to address the emerging challenges of agility and resourcefulness.

Despite its recognized significance in fostering innovation at the micro level, scholarly understanding of the antecedents and underlying psychological mechanisms that stimulate employee engagement in bootlegging innovation behavior remains fragmented and underdeveloped (Globocnik et al., 2022; J. Zhang et al., 2025). In particular, the role of leadership behavior in promoting such informal innovation efforts remains unclear. Although various leadership styles—such as transformational leadership, empowering leadership, and ethical leadership—have been widely examined in the innovation literature, coaching leadership represents a particularly relevant leadership approach for explaining employees’ engagement in informal and self-initiated innovation activities. Unlike traditional leadership styles that primarily rely on inspiration, vision articulation, or authority delegation, coaching leadership emphasizes developmental interaction, individualized guidance, and continuous feedback aimed at unlocking employees’ potential and fostering long-term capability development. Through goal clarification, reflective dialogue, and supportive mentoring, coaching leaders actively facilitate employees’ self-discovery and competence development, thereby cultivating a stronger sense of autonomy and personal growth in the workplace. New insights in contemporary leadership research draw attention to coaching leadership, a relationship-based and development-oriented leadership style that is a potential influencing factor in shaping employees’ autonomous behavior (Owens & Hekman, 2012). Coaching leadership is characterized by behaviors that prioritize employee growth, including offering guidance, facilitating autonomous problem-solving, empowering individual potential, and providing both cognitive and emotional support in the face of work-related challenges (Jong, 2007; Robinson, 2024). Leaders who adopt a coaching style are likely to create psychologically safe environments in which employees perceive a lower risk of interpersonal threats when engaging in exploratory, boundary-pushing, and potentially unorthodox innovation activities (Neves, 2024). Such a leadership climate is particularly conducive to bootlegging innovation behavior, which inherently involves stepping beyond the parameters of formal job descriptions and challenging the status quo without prior approval.

Although the positive link between coaching leadership and innovative work behavior has been conceptually recognized, the psychological process of translating coaching leadership into specific informal innovative outcomes still lacks sufficient theoretical and empirical validation (X. Zhang, 2020). To address this gap, the present study proposes that coaching leadership enhances employees’ intrinsic motivation by cultivating meaning in work. Work meaning, defined as an employee’s subjective perception that their work is purposeful, valuable, and socially significant, is a pivotal cognitive lens through which employees interpret their daily tasks (Cheney et al., 2008; Lysova et al., 2023). Employees who perceive their work as meaningful are more likely to engage in discretionary behaviors that transcend formal role expectations, including initiating and pursuing innovative ideas without explicit managerial endorsements (Walumbwa et al., 2010). Moreover, we integrate insights from the organizational psychological ownership literature, arguing that meaningful work experiences foster a deeper sense of psychological ownership over one’s tasks and the broader organization. Organizational psychological ownership, conceptualized as a feeling of possessiveness and psychological attachment to an organization, can motivate employees to devote additional cognitive, emotional, and behavioral resources to organizational well-being (Van Dyne & Pierce, 2004), including proactive engagement in innovation efforts, such as bootlegging. By synthesizing leadership theory and the construct of organizational psychological ownership, this study proposes a comprehensive and integrative model that explicates the indirect and sequential pathways through which leadership coaching facilitates bootlegging innovation behavior (Huang et al., 2022). In doing so, this study aims to enrich the theoretical conversation surrounding informal innovation and contribute meaningfully to the emerging discourse on the mechanisms through which leadership shapes proactive, self-directed employee behaviors in increasingly complex and dynamic organizational environments.

Building on these perspectives, this study seeks to advance understanding of how leadership fosters informal and self-initiated innovation by focusing on coaching leadership as a key antecedent of employees’ bootlegging innovation behavior. While prior research has acknowledged the role of leadership in shaping innovative work behavior, little is known about the psychological mechanisms through which coaching-oriented leadership translates into employees’ engagement in unofficial innovation activities. To address this gap, this study develops and empirically tests a serial mediation model in which work meaning and organizational psychological ownership sequentially transmit the effects of coaching leadership on bootlegging innovation behavior. By integrating leadership theory with the studies on meaningful work and psychological ownership, this study offers a more nuanced account of how leadership influences employees’ motivation and sense of responsibility underlying informal innovation. In doing so, this research contributes to the growing body of work on employee-driven innovation by highlighting the indirect and process-oriented role of coaching leadership in shaping proactive, organization-oriented behaviors beyond formal role expectations.

## 2. Theory and Hypotheses

### 2.1. Coaching Leadership and Bootlegging Innovation Behavior

Coaching leadership, an emerging form of relational leadership, promotes employees’ discretionary and proactive behaviors within organizational contexts (Cui et al., 2022). Unlike traditional directive leadership styles, coaching leadership emphasizes individualized support, continuous feedback, and cultivation of self-efficacy among employees. By fostering developmental relationships and encouraging self-reflection, coaching leaders enable employees to recognize and leverage their potential, which provides a psychological foundation for voluntary engagement in innovation-related activities (Hoßbach et al., 2024). Through consistent guidance and encouragement, coaching leaders help employees internalize both task-related and growth-oriented goals, thereby motivating them to pursue creative endeavors beyond the boundaries of formal job descriptions.

One prominent innovation-related behavior influenced by coaching leadership is bootlegging innovation behavior, which refers to employees’ self-initiated and sometimes covert pursuit of new ideas, projects, or solutions without prior formal approval (Surma-aho et al., 2024; Guarana & Avolio, 2022). Bootlegging innovation behavior represents an important avenue through which employees overcome organizational constraints to explore novel opportunities. This behavior typically involves significant risk as it occurs outside official work assignments, often using individual or unallocated organizational resources. Coaching leadership, by cultivating psychological safety and signaling tolerance for experimentation, reduces employees’ perceived risks associated with informal innovation efforts (Edmondson, 2018). Employees under coaching leaders are more likely to believe that their leaders will interpret proactive, even unauthorized, innovation efforts as a sign of commitment and initiative rather than deviance, thereby lowering social and organizational barriers to bootlegging innovation behavior (Wang et al., 2023).

Furthermore, coaching leadership creates an environment that nurtures intrinsic motivation, which is highly relevant for encouraging bootlegging innovation behavior (Globocnik, 2023). Employees who perceive that their leader is genuinely interested in their individual development and supportive of learning from failures are more inclined to engage in explorative and experimental behaviors that are characteristic of bootlegging. This leadership style provides cognitive and emotional resources such as confidence and encouragement, which help employees cope with the uncertainties and setbacks often encountered during bootlegging innovation behavior (Yuan & Woodman, 2010; Huang et al., 2022). In addition, coaching leaders often grant greater autonomy in task execution and decision making, which indirectly legitimizes employees’ sense of ownership over their ideas and motivates them to invest time and effort in developing these ideas, even without explicit supervisory consent (Matei & Veith, 2023).

The relationship between coaching leadership and bootlegging innovation behavior can be explained through self-determination theory and social exchange theory. Self-determination theory suggests that individuals are more likely to engage in proactive and innovative behaviors when their psychological needs for autonomy, competence, and relatedness are satisfied (Deci & Ryan, 2000). Coaching leadership emphasizes individualized support, developmental feedback, and empowerment, which enhance employees’ intrinsic motivation and confidence to explore new ideas. Such support encourages employees to engage in exploratory activities beyond formal job roles, including bootlegging innovation behavior. In addition, social exchange theory posits that employees tend to reciprocate supportive leadership with positive behaviors that benefit the organization (Blau, 1964). When coaching leaders demonstrate trust and invest in employees’ development, employees feel obligated to repay this support by contributing additional effort and innovative initiatives. Consequently, coaching leadership creates both motivational and relational conditions that facilitate employees’ engagement in bootlegging innovation behavior.

Empirical studies confirmed the link between supportive leadership behaviors and employee engagement in bootlegging innovation behavior. For instance, J. Zhang et al. (2025) found that leader behaviors that emphasize individual growth and proactive motivation significantly predict employees’ bootlegging innovation behavior across knowledge-intensive industries. By reinforcing a growth mindset and reducing the perceived penalties for failure, coaching leadership effectively nurtures the cognitive and affective conditions essential for bootlegging (Wefald, 2024). Taken together, these arguments suggest that coaching leadership establishes a developmental and psychologically safe context that encourages employees to engage in bootlegging innovation behavior. Accordingly, the following hypothesis is proposed:

**H1.**  *Coaching leadership has a positive effect on bootlegging innovation behavior.*

### 2.2. Mediating Effect of Work Meaning

Coaching leadership, as a relational and developmental style, concentrates on facilitating employee growth, self-directed learning, and autonomous problem-solving capabilities. Leaders who engage in coaching behaviors typically provide individualized support, constructive feedback, and purposeful guidance (Milner et al., 2018; Toni et al., 2025), all of which contribute to creating an environment where employees perceive their work as valuable, significant, and aligned with their individual aspirations and organizational objectives. Through such leader-employee interactions, coaching leadership enhances the psychological salience of work, allowing employees to experience a deeper sense of purpose and stronger identification with their professional roles.

This perceived meaning in work is a crucial psychological mechanism through which coaching leadership influences employee behavior, particularly in the context of proactive, discretionary innovation efforts, such as bootlegging innovation behavior. When employees experience their work as meaningful, they are more likely to transcend formal job descriptions and prescribed organizational routines (Bailey & Madden, 2017), exhibiting greater initiative, resilience, and creative engagement in the pursuit of novel solutions (Malik & Garg, 2020). Specifically, the experience of work meaning has been shown to strengthen employees’ intrinsic motivation, thus fostering a cognitive and emotional commitment that drives them to invest time and effort in activities they perceive as valuable, even in the absence of explicit managerial approval or formal reward structures (Steger et al., 2012).

In this regard, work meaning acts as an explanatory link between coaching leadership and bootlegging innovation behavior (L. Zhang et al., 2023). Employees who perceive their work as meaningful are more inclined to interpret innovative problem solving as a natural extension of their role responsibility rather than as a risk-laden deviation from organizational norms. Consequently, these employees demonstrate a heightened willingness to engage in bootlegging innovation behavior, the self-initiated, often covert pursuit of innovative ideas as expressions of both individual fulfillment and perceived obligation toward organizational improvement (Nanyangwe et al., 2021). Therefore, the influence of coaching leadership on bootlegging innovation behavior is not direct or automatic, but operates through employees’ perceptions of work meaning.

By embedding meaning into employees’ daily work experiences, coaching leaders indirectly stimulate an internal motivational state that propels employees to engage in bootlegging innovative behaviors beyond formal expectations, even in the absence of immediate external validation or support. Taken together, these theoretical arguments and empirical findings suggest that work meaning serves as a key psychological mechanism linking coaching leadership to employees’ bootlegging innovation behavior. Accordingly, the following hypothesis is proposed:

**H2.**  *Work meaning mediates the relationship between coaching leadership and bootlegging innovation behavior.*

### 2.3. Mediating Effect of Organizational Psychological Ownership

Coaching leadership emphasizes developmental support, empowerment, and individualized guidance, cultivating an interpersonal environment wherein employees are more likely to develop a profound psychological attachment to the organization (Toni et al., 2025). By providing constructive feedback, facilitating self-reflection, and encouraging autonomous problem-solving, coaching leaders signal trust and recognition of employees’ contributions. These leadership practices create conditions that foster a sense of belonging and perceived organizational inclusion, which are essential precursors to the formation of organizational psychological ownership. As employees increasingly internalize an organization’s goals and perceive its success as personally meaningful, the likelihood of experiencing organizational psychological ownership increases significantly (Pierce et al., 2001; Kim & Beehr, 2017). Once organizational psychological ownership is established, it becomes a motivational mechanism that propels employees toward discretionary and proactive behaviors, including bootlegging innovation. Employees who perceive the organization as “theirs” are intrinsically motivated to safeguard and enhance its welfare, even in the absence of explicit managerial directives or formal recognition. This psychological state prompts employees to go beyond prescribed roles, investing individual time and resources in innovative initiatives that they believe will yield organizational benefits (Cascio & Boudreau, 2010). In this context, bootlegging innovation behavior, though typically characterized by its unsanctioned or informal nature, emerges as an expression of employees’ self-driven commitment to organizational advancement, rather than as behaviors of deviance or opportunism.

Furthermore, the mediating role of organizational psychological ownership elucidates the psychological processes through which coaching leadership fosters bootlegging innovation behavior (Hao et al., 2024). By granting autonomy, acknowledging individual capabilities, and nurturing individual growth, coaching leadership activates employees’ intrinsic needs for efficacy, self-determination, and meaningful affiliation (Alshahrani et al., 2025). Once fulfilled, these needs form the foundation of psychological ownership. Employees who perceive themselves as integral to the organization’s present and future are more inclined to assume individual responsibility for their outcomes (McShane & Cunningham, 2012; Glavas & Kelley, 2014). Consequently, this sense of responsibility manifests in behaviors aimed at initiating and implementing innovative solutions, often without awaiting formal approval. The emergence of bootlegging innovation behavior under such conditions reflects employees’ proactive efforts to translate organizational psychological ownership into tangible contributions, particularly when conventional organizational channels are perceived as slow, risk-averse, or unsupportive of novel ideas.

This mediating pathway also highlights that the relationship between coaching leadership and bootlegging innovation behavior is unlikely to be direct or automatic. Rather, the emergence of bootlegging innovation behavior depends on the extent to which coaching leadership practices succeed in engendering organizational psychological ownership among employees (Salleh & Rezal, 2021). Without feelings of ownership, even the most supportive and empowering leadership may fail to elicit the initiative and commitment necessary for bootlegging innovation to materialize. Conversely, when employees experience a strong sense of ownership, even subtle or indirect expressions of coaching leadership can activate self-motivated behaviors aimed at improving the organization, including the pursuit of unsanctioned innovative activities (Goenaga, 2024). Organizational psychological ownership is an important psychological channel for transforming leadership behavior into proactive innovation achievements. In addition, this perspective underscores that bootlegging innovation behavior is not merely the result of individual dispositional tendencies or situational constraints but is significantly shaped by the social and psychological environment fostered by leadership practices. Coaching leadership lays the groundwork for a relational climate in which employees feel entrusted, valued, and empowered (Torres, 2024). This climate strengthens the employee-organization bond, reinforcing the psychological state of ownership. Consequently, this psychological state fuels the willingness to engage in innovative efforts that may fall outside conventional boundaries. Employees are thus more likely to initiate bootlegging innovation behavior not as behavior of resistance or rebellion but as constructive and intentional endeavor to serve the interests of the organization, reflecting the internalization of organizational objectives as self-relevant goals. Coaching leadership facilitates psychological ownership by fostering autonomy, self-worth, and a sense of inclusion (Bai et al., 2024), which subsequently encourages employees to engage in bootlegging innovation as expression of their commitment to organizational success. Thus, psychological ownership serves as a pivotal psychological link that transforms leadership influence into innovative action, providing a nuanced understanding of how and why leadership coaching contributes to the emergence of bootlegging innovation within organizational contexts. Therefore, the following hypothesis is proposed:

**H3.**  *Organizational psychological ownership mediates the relationship between coaching leadership and bootlegging innovation behavior.*

### 2.4. The Chained Mediating Role of Work Meaning and Organizational Psychological Ownership

Coaching leadership creates a workplace atmosphere characterized by empowerment, support, and developmental focus, which stimulates employees to experience heightened work meaning (Lee et al., 2019). Leaders who adopt a coaching style tend to encourage employees to reflect on their roles and contributions in a broader organizational context (Milner et al., 2018). Employees will then perceive their work as purposeful, valuable, and aligned with their individual and collective goals, which increases their intrinsic motivation and leads them to go beyond formal job requirements and engage in self-initiated innovative activities, including bootlegging innovation. However, this relationship is not immediate; rather, it unfolds through a deeper psychological process that includes the development of organizational psychological ownership.

The experience of work meaning derived from coaching leadership strengthens employees’ emotional and cognitive connections with their organizations, subsequently fostering a sense of organizational psychological ownership (Dawkins et al., 2017). Employees who see their work as meaningful internalize the organization’s goals and values, gradually building a mental framework in which the organization is no longer seen as an external entity, but rather as something to which they psychologically belong and feel responsible (Masterson & Stamper, 2003). This sense of ownership is crucial because it cultivates intrinsic motivation to invest effort and take initiative for the organization’s benefit, even when such actions occur outside of formal approval channels.

As organizational psychological ownership develops, employees are more likely to display bootlegging innovation behavior as an expression of their commitment and responsibility toward the organization (Jia et al., 2023). The sense of ownership motivates employees to pursue innovative ideas they believe will advance organizational success, even without explicit managerial endorsement or resources. Thus, organizational psychological ownership serves as a motivational engine that transforms the intrinsic desire to contribute to bootlegging innovation behavior (Liu et al., 2019). Employees who feel ownership over their organization are less constrained by formal rules and procedures when they encounter opportunities for improvement or innovation, as they view such initiatives as an extension of their role and responsibility as “owners” of the organization. Thus, coaching leadership initiates a developmental process where the leader’s support first fosters work meaning, which, in turn, enhances organizational psychological ownership and, ultimately, promotes bootlegging innovation behavior (Criscuolo et al., 2014; Wang et al., 2023). This sequential mediation suggests that the pathway from leadership style to employee innovation is neither linear nor superficial but rather embedded in a chain of cognitive and affective mechanisms. Coaching leadership does not directly push employees toward bootlegging innovation behavior; instead, it creates psychological conditions that make such behaviors more likely and meaningful to employees (Guarana & Avolio, 2022).

Furthermore, the sequential nature of mediation highlights the importance of work meaning and organizational psychological ownership as complementary mechanisms. Work meaning primarily addresses the cognitive interpretation and emotional evaluation of one’s tasks, whereas organizational psychological ownership focuses on a deeper relational bond with the organization (Y. Zhang et al., 2021). Without work meaning, employees may not develop the individual engagement required to feel organizational psychological ownership (Chai et al., 2020). Similarly, even if employees find their work meaningful, the absence of organizational psychological ownership may limit their willingness to engage in discretionary and potentially risky behaviors such as bootlegging innovation. The combination of both mechanisms ensures that employees not only understand the importance of their work but also internalize a sense of stewardship over the organization’s future, which drives them to act innovatively, even under informal or unapproved conditions. This mediating chain also reveals the nuanced role of leadership in shaping innovative behavior. Coaching leadership does not directly dictate outcomes but instead enables psychological states that guide employees toward proactive, self-directed innovation (Masood et al., 2024). Emphasis on employee development, individual growth, and meaningful work experiences creates an environment in which ownership and responsibility are self-generated rather than externally imposed (Gardner & Schermerhorn, 2004). This self-generated motivation is particularly important in explaining bootlegging innovation behavior, as such behavior often occurs in the absence of formal recognition, support, or rewards. Employees who feel both the meaningfulness of their work and a strong sense of ownership are more willing to take risks, invest effort, and persevere in innovative pursuits, even when these actions involve navigating formal systems or taking initiative on their own (Steger, 2016).

**Figure 1:**
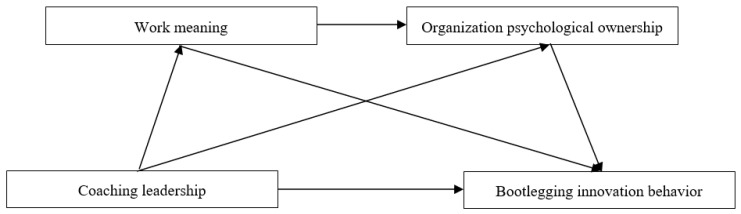
Research model.

In summary, the relationship between coaching leadership and bootlegging innovation behavior is best understood as a sequential psychological process involving work meaning and organizational psychological ownership. Coaching leadership first enhances employees’ perceptions of work meaning, shaping how they cognitively and emotionally interpret the significance of their work. This heightened sense of meaning subsequently facilitates the development of organizational psychological ownership, as employees internalize organizational goals and perceive a stronger sense of responsibility toward the organization. In turn, this ownership-oriented psychological state motivates employees to engage in bootlegging innovation behavior as a proactive and organization-oriented form of discretionary innovation. This sequential chain of influence underscores that the effect of coaching leadership on bootlegging innovation behavior is neither direct nor instantaneous, but unfolds through interconnected motivational and ownership-based psychological mechanisms that drive employees’ self-initiated innovative behaviors beyond formal role expectations. Accordingly, we propose the following hypothesis:

**H4.**  *Work meaning and organizational psychological ownership mediate the relationship between coaching leadership and bootlegging innovation behavior in turn (Figure 1).*

## 3. Methods

This study conducted a questionnaire survey among employees working in 14 high-tech enterprises located in Shandong and Henan provinces in China. The data collection was carried out from September to November 2025, targeting organizations operating in the manufacturing industry.

A total of 500 questionnaires were distributed, and 427 valid responses were retained for analysis, yielding an effective response rate of 85.40%. During the data cleaning process, questionnaire quality was evaluated based on response completeness, response patterns, and completion time. Questionnaires containing missing values on key variables, exhibiting clearly patterned responses, or showing abnormally short completion times were excluded to ensure the reliability and validity of the dataset.

Regarding sample recruitment, this study employed a convenience sampling approach. After obtaining organizational access through alumni networks and professional contacts, questionnaires were distributed to eligible employees via an online survey platform. To be eligible, respondents had to be currently employed and capable of understanding and responding to questions related to coaching leadership, work meaning, organizational psychological ownership, and bootlegging innovation behavior. In organizations where multiple employees participated, respondents were recruited from different positions whenever possible to avoid collective responses or proxy completion. Because the focal constructs involve employees’ perceptions of leadership and their own innovative behaviors, employees themselves were considered the most appropriate informants. The survey instructions emphasized that responses should be based on respondents’ own work experiences and completed independently.

With regard to research ethics, the study followed standard ethical principles for social science research. Before completing the questionnaire, respondents were informed of the purpose of the study, the anonymity and confidentiality of their responses, and the voluntary nature of participation. Participants were informed that they could withdraw at any time without any negative consequences. No personally identifiable information was collected, and the data were used solely for academic research purposes. According to the research management requirements of the authors’ institution, the study was classified as a low-risk anonymous questionnaire survey, and therefore formal ethics approval was not required.

### 3.1. Measures

To test the hypothesized relationships empirically, we administered a structured questionnaire survey to collect data on the key constructs of interest. To ensure reliability and validity, all measurement items were adapted from scales established in previous studies. The questionnaire used a 5-point Likert scale, where 1 = strongly disagree, 2 = disagree, 3 = average, 4 = agree, and 5 = completely agree.

Coaching leadership. We used the 12-item scale developed by Anderson (2013). Some examples of the items are “I have helped them develop themselves as individuals,” “I am very good at observing their work to guide how I manage them,” and “If any of them has a good idea, I always use it.” The Cronbach’s alpha was 0.915.

Work meaning. We used the three-item scale developed by Spreitzer (1995). The items include “The work I do is very important to me,” “My job activities are personally meaningful to me,” and “The work I do is meaningful to me.” Employees completed a part of the questionnaire. Higher scores indicated a stronger sense of work meaning. The Cronbach’s alpha was 0.790.

Organizational psychological ownership. We used four items taken from (Brown et al., 2014). The items include “I feel a very high degree of personal ownership for this job,” “I sense that this is my job,” “I feel a very high degree of personal ownership for the work that I do,” and “The work I do at this organization is mine.” The Cronbach’s alpha was 0.834.

Bootlegging innovation behavior. We used the four-item scale developed by Criscuolo et al. (2014). The items include “I have the flexibility to work my way around my official work plan, digging into new potentially valuable business opportunities,” “My work plan does not allow me the time to work on anything other than the projects I have been assigned to,” “I am running several pet projects that allow me to learn about new areas,” and “I proactively take time to work on unofficial projects to seed future official projects.” The Cronbach’s alpha was 0.841.

### 3.2. Control Variables

The respondents’ gender, age, educational background, and tenure were used as control variables. Therefore, demographic variables, such as gender, age, and educational background, were used as control variables in the empirical analysis. Gender was coded as 1 for males and 2 for females. Age was measured in years, and education was measured as the years in which the participant completed that stage of education.

## 4. Results

### 4.1. Descriptive Analysis

We performed descriptive statistical analyses for the demographic variables based on data collected from 427 participants. Of the participants, 55.04% were male and 44.96% were female. In terms of age, participants aged under 25 years accounted for the highest proportion (30.44%), those aged 26–30 accounted for 24.12%, those aged 31–35 accounted for 11.94%, those aged 36–50 accounted for 26.23%, and those aged over 51 years accounted for the lowest proportion (7.26%). Regarding academic qualifications, the proportion of participants with high school or technical secondary school and college education was the highest at 29.51%. Meanwhile, those with a master’s degree comprise only 7.49% of the sample. Regarding tenure, 1–5 years accounted for the highest proportion (53.16%). 6–10 years accounted for 13.11%, 11–15 years accounted for the lowest proportion (7.73%), 16–20 years accounted for 12.65%, and over 21 years accounted for 13.35%. In terms of position level, 198 respondents (46.37%) were frontline employees, 72 (16.86%) were junior managers, 56 (13.11%) were middle managers, 18 (4.22%) were senior managers, and 83 (19.44%) fell into other categories. Regarding functional roles, the sample included respondents from general management (19.20%), administration/human resources (*n* = 73, 17.10%), sales/marketing (*n* = 64, 14.99%), worker/service positions (*n* = 57, 13.35%), planning/advertising (*n* = 40, 9.37%), research and development (*n* = 39, 9.13%), technology/IT (*n* = 28, 6.56%), finance/accounting (*n* = 24, 5.62%), and production (*n* = 20, 4.68%). The respondents also came from organizations of diverse sizes, including firms with fewer than 50 employees (41.45%), 51–100 employees (14.29%), 101–200 employees (9.60%), 201–500 employees (11.48%), and more than 500 employees (23.19%). In terms of organizational ownership, the sample was drawn mainly from private enterprises (40.75%) and state-owned enterprises (13.11%), while also including foreign-invested firms, joint ventures, and other organizations.

### 4.2. Confirmatory Factor Analysis and Reliability Analysis

We used a confirmatory factor analysis to test the model fit. The results were as follows: χ^2^ (*p*) = 262.398 (0.040), χ^2^/df = 1.171, RMSEA = 0.020, CFI = 0.992, TLI = 0.991, and SRMR = 0.029. The fitting index of the model exhibited a good effect. Although the chi-square test results (χ^2^ = 262.398, *p* = 0.040) showed a significant difference in the model fit, the chi-squared/degrees-of-freedom ratio (χ^2^/df = 1.171) indicated that it was acceptable, considering the effects of large samples. Specifically, the structural model explained 43.8% of the variance in work meaning (R^2^ = 0.438), 89.9% of the variance in organizational psychological ownership (R^2^ = 0.899), and 36.6% of the variance in bootlegging innovation behavior (R^2^ = 0.366). These results provide a clearer indication of the model’s explanatory power for each endogenous construct.

The average variance extracted (AVE) and composite reliability (CR) were analyzed. The AVE value measures the proportion of explanatory variance for each indicator in the construct (Hair et al., 2017). The values for each variable were 0.474 for coaching leadership, 0.557 for work meaning, 0.556 for organizational psychological ownership, and 0.572 for bootlegging innovation behavior. Although the AVE value of coaching leadership is less than 0.5, it is still acceptable because of a CR of more than 0.7 (Bagozzi & Yi, 1988). The other values were greater than 0.5, indicating good convergent validity.

The CR value measures a construct’s internal consistency (Sen, 1993). The CR values for each variable were 0.915 for coaching leadership, 0.790 for work meaning, 0.834 for organizational psychological ownership, and 0.842 for bootlegging innovation behavior. All values were greater than 0.7, indicating good confidence. Confirmatory factor analysis (CFA) was conducted on the four proposed construct models to assess the validity and reliability of the constructs. Table 1 shows the CFA results with satisfactory model fit indices (χ^2^/df = 1.171, *p* < 0.001; CFI = 0.992; TLI = 0.991; RMSEA = 0.020; SRMR = 0.029). We summarize these results in Table 1.

### 4.3. Correlation Analysis

The mean values for coaching leadership, work meaning, organizational psychological ownership, and bootlegging innovation behavior were 3.688, 3.808, 3.748, and 3.697, respectively. The standard deviations (SD) of coaching leadership, work meaning, organizational psychological ownership, and bootlegging innovation behavior were 0.626, 0.725, 0.728, and 0.751, respectively. Coaching leadership was associated with work meaning (r = 0.563, *p* < 0.001), organizational psychological ownership (r = 0.563, *p* < 0.001), and bootlegging innovation behavior (r = 0.467, *p* < 0.001). Work meaning was positively correlated with organizational psychological ownership (r = 0.769, *p* < 0.001) and bootlegging innovation behavior (r = 0.466, *p* < 0.001). Organizational psychological ownership is positively correlated with bootlegging innovation behavior (r = 0.459, *p* < 0.001). We provide these results in Table 2.

### 4.4. Chained Mediation Analysis

We applied the M-plus serial multiple mediation model to examine how the variables used in this study interacted. To test the indirect effects of the chained mediation analysis, we used the bootstrap method to perform an analysis using the M-plus 8.3 statistical software. If the upper and lower bounds of the coefficients obtained in the middle of the 95% confidence interval (CI) do not include 0, then we can consider them as significant (Lee et al., 2019). The results of the path analysis and standardized effects are listed in Table 3 and Table 4, unstandardized effects are listed in Table 5 and Table 6. The standardized path analysis results showed that coaching leadership had a direct positive effect on bootlegging innovation behavior (β = 0.217, *p* < 0.001, CI: 0.105–0.330), thus supporting Hypothesis 1. Coaching leadership also had a direct positive effect on work meaning (β = 0.530, *p* < 0.001, CI: 0.447–0.603), and organizational psychological ownership (β = 0.193, *p* < 0.001, CI: 0.109–0.275). Work meaning had a direct positive effect on organizational psychological ownership (β = 0.633, *p* < 0.001, CI: 0.553–0.703) and bootlegging innovation behavior (β = 0.158, *p* < 0.05, CI: 0.033–0.285). Organizational psychological ownership had a direct positive effect on bootlegging innovation behavior (β = 0.204, *p* < 0.01, CI: 0.078–0.334). The results of the standardized bootstrap method showed that coaching leadership → work meaning → bootlegging innovation behavior. We found a positive mediating effect on bootlegging innovation behavior (β = 0.091, CI: 0.019–0.168), thereby supporting Hypothesis 2. The positive mediating effect of coaching leadership → organizational psychological ownership → bootlegging innovation behavior (β = 0.043, CI: 0.016–0.083) was also confirmed, supporting Hypothesis 3. In addition, the sequential continuous positive mediating effect of coaching leadership → work meaning → organizational psychological ownership → bootlegging innovation behavior (β = 0.075, CI: 0.029–0.130) did not include 0 at the 95% confidence level. Work meaning and organizational psychological ownership mediate the relationship between coaching leadership and bootlegging innovation behavior. Coaching leadership facilitates work meaning and organizational psychological ownership, while organizational psychological ownership encourages bootlegging innovation behavior, supporting Hypothesis 4. The findings support a partial mediation model rather than a full mediation model. Specifically, work meaning and organizational psychological ownership significantly mediate the relationship between coaching leadership and bootlegging innovation behavior, while the direct effect of coaching leadership on bootlegging innovation behavior remains significant. This indicates that the effect of coaching leadership on bootlegging innovation behavior is only partially transmitted through the proposed mediators. This result is theoretically reasonable because bootlegging innovation behavior is a complex and risky form of informal innovation that is unlikely to be fully explained by a single set of psychological mechanisms. Although other mechanisms, such as psychological empowerment and psychological safety, may also be relevant, they were not included in the present model because this study was designed to focus on a more specific explanatory path. Work meaning captures employees’ perception of the value and purpose of their work, whereas organizational psychological ownership reflects their sense of possession and responsibility toward the organization. These two mechanisms are especially relevant for explaining why coaching leadership may promote bootlegging innovation behavior. Therefore, this study deliberately focused on the mediating roles of work meaning and organizational psychological ownership to preserve theoretical clarity and model parsimony, while recognizing other psychological mechanisms as important directions for future research.

To further examine the robustness of the proposed serial mediation structure, we compared the explanatory power of the proposed model (CL → WM → OPO → BIB) with a reverse-order model (CL → OPO → WM → BIB). As shown in Table 7, the proposed model demonstrates stronger explanatory power for organizational psychological ownership (R^2^ = 0.568) compared with the reverse-order model (R^2^ = 0.280), whereas the reverse-order model explains more variance in work meaning (R^2^ = 0.568) than the proposed model (R^2^ = 0.281). Importantly, both models explain the same level of variance in bootlegging innovation behavior (R^2^ = 0.245). Taken together, these findings provide empirical support for retaining the theoretically proposed mediator sequence from work meaning to organizational psychological ownership.

## 5. Conclusions

### 5.1. Discussion

This study examines how coaching leadership promotes employees’ bootlegging innovation behavior by elucidating the sequential mediating roles of work meaning and organizational psychological ownership. The findings provide empirical support for the proposed model and demonstrate that coaching leadership is positively associated with employees’ engagement in bootlegging innovation. This result aligns with prior research suggesting that developmental and supportive leadership behaviors foster employee initiative and exploration beyond formal role expectations. By emphasizing guidance, feedback, and employee development, coaching leadership appears to create a psychological climate in which employees feel encouraged to pursue innovative ideas even when such activities are not formally sanctioned by the organization.

Previous studies have shown that supportive and developmental leadership styles, such as coaching, transformational, and empowering leadership, are positively related to employees’ proactive and innovative behaviors (Agarwal et al., 2012). Leaders who provide developmental feedback and encourage autonomy can enhance employees’ psychological resources and intrinsic motivation, thereby stimulating innovative work behavior. Similarly, research on bootlegging innovation indicates that employees are more likely to engage in such informal innovation when they perceive organizational support, psychological safety, or strong organizational identification (Criscuolo et al., 2014; Globocnik & Salomo, 2015). However, most prior studies have focused on direct relationships or single mediating mechanisms. Extending this line of research, the present study reveals a more nuanced psychological pathway by identifying work meaning and organizational psychological ownership as sequential mediators linking coaching leadership to bootlegging innovation behavior. These findings not only support the positive role of developmental leadership in promoting employee innovation but also deepen understanding of how employees’ cognitive interpretations of work and sense of ownership jointly drive informal innovation.

More importantly, the findings indicate that the influence of coaching leadership on bootlegging innovation behavior operates primarily through employees’ internal psychological processes rather than through a purely direct effect. Specifically, coaching leadership enhances employees’ perceptions of work meaning, which subsequently strengthens their sense of organizational psychological ownership and motivates engagement in bootlegging innovation. This sequential mediation suggests that employees are more likely to engage in informal and discretionary innovation when they perceive their work as meaningful and feel a sense of responsibility toward the organization. In this regard, work meaning functions as a foundational cognitive and motivational mechanism that shapes how employees interpret their roles, while organizational psychological ownership transforms this interpretation into a deeper sense of commitment and stewardship toward organizational outcomes.

This study offers a process-oriented explanation of how leadership behaviors translate into informal innovation outcomes. The findings suggest that bootlegging innovation should not be viewed merely as deviant or opportunistic behavior but as a constructive and organization-oriented response rooted in employees’ intrinsic motivation and ownership perceptions. Overall, this study extends existing leadership and innovation research by highlighting the indirect and sequential psychological pathways through which coaching leadership fosters employee-driven innovation. The results underscore the importance of considering employees’ subjective work experiences and ownership perceptions when examining how leadership practices shape proactive and informal innovation behaviors in contemporary organizational contexts.

### 5.2. Theoretical Implications

Collectively, this study contributes to the leadership and innovation literature by clarifying the psychological pathways through which coaching leadership fosters informal, employee-driven innovation. This study offers several meaningful theoretical contributions to the fields of leadership and organizational behavior by illuminating the underlying mechanisms through which coaching leadership fosters bootlegging innovation behavior. Prior research acknowledged the significance of leadership styles in shaping various forms of employee behavior, especially those associated with creativity, proactivity, and performance. However, most of the existing literature focused on formalized innovation efforts sanctioned by the organization and embedded in predefined roles or processes. By contrast, the concept of bootlegging highlights a distinctive form of innovative behavior characterized by its unofficial, autonomous, and self-initiated nature, which, despite lacking formal authorization, often produces valuable outcomes for organizational development (Globocnik et al., 2022). By linking coaching leadership to bootlegging innovation behavior, this study expands the existing theoretical frameworks on leadership and innovation by shedding light on how leaders can encourage and facilitate employees’ informal and discretionary innovative efforts, which are typically overlooked in mainstream innovation research.

The existing leadership literature is increasingly moving away from leader-centred views toward a more employee-centred understanding of how leadership exerts its influence (Cardon, 2019). In line with this emerging perspective, this study suggests that the effectiveness of coaching leadership in stimulating bootlegging innovation behavior is not a direct or automatic process. Rather, it operates through the cultivation of employees’ positive psychological states. Specifically, the identification of work meaning as a mediator underscores the idea that when coaching leaders help employees find purpose, value, and individual significance in their daily tasks, it enhances their willingness to engage in self-directed innovative actions, even when these actions fall outside formal job descriptions or organizational mandates (Artis & Harris, 2007). This finding contributes to the literature by empirically validating the importance of work meaning in connecting leadership practices with proactive innovation behavior.

Overall, this study makes a novel contribution by bridging the gap between leadership theory and innovation literature through the lens of bootlegging innovation behavior, which has been relatively underexplored in prior studies. It moves beyond traditional views of innovation as a formalized, top-down process and highlights the role of employees’ internal motivation and sense of agency in driving innovation from the bottom up. In doing so, this study enriches theoretical conversation on both the antecedents and mechanisms of bootlegging innovation behavior. It also offers a valuable psychological perspective on how leadership can foster an environment in which employees are motivated to take initiative, experiment with new ideas, and engage in constructive deviance to benefit the organization.

### 5.3. Practical Implications

The findings of this study offer significant practical implications for organizations aiming to cultivate an environment conducive to innovation, particularly proactive, self-initiated, and potentially rule-bending innovative behaviors encapsulated in the concept of bootlegging. Given the increasingly volatile nature of contemporary business environments, organizations require not only formal innovation programs but also employees to engage in spontaneous and self-directed innovation (Rana et al., 2016). This study highlights the role of coaching leadership in facilitating such behaviors, both directly and through the enhancement of employees’ work meaning and organizational psychological ownership.

First, the positive association between coaching leadership and bootlegging innovation behavior underscores the critical role leadership style plays in shaping employee behaviors that extend beyond formalized work expectations (Wang et al., 2023). Coaching leadership, characterized by individualized guidance, empowerment, and the cultivation of employees’ professional growth, creates a sense of psychological safety and fosters intrinsic motivation that embolden employees to pursue innovative ideas (Gilliam, 2024), even when these ideas may deviate from conventional processes or lie outside the scope of their formal job descriptions. Leaders with a coaching orientation are more likely to engage in open dialogue with employees, encourage risk-taking, and support learning from failure, all of which are essential conditions for bootlegging innovation behavior to occur (Globocnik et al., 2022). As such, organizations aiming to foster innovative behavior should invest in leadership development programs that emphasize coaching competencies, including active listening, feedback provision, developmental support, and the articulation of a compelling vision that frames individual work as meaningful within a broader organizational context.

Additionally, the mediating role of work meaning in the relationship between coaching leadership and bootlegging innovation behavior indicates that cultivating a sense of work meaning can promote employee innovation competence (Nanyangwe et al., 2021). When employees perceive their work as purposeful, they are more likely to transcend role boundaries and invest cognitive, emotional, and behavioral resources in innovative solutions, even without explicit managerial mandates or organizational sanctions (Iqbal et al., 2023). Coaching leaders, by aligning individual aspirations with organizational goals, play a central role in shaping perceptions of meaningfulness (Göçen, 2021). Practical initiatives, such as job crafting workshops, purpose-driven team meetings, and leader-employee developmental dialogues, may further amplify this effect, creating a virtuous cycle in which meaningful work fosters bootlegging innovation, which, in turn, reinforces an employee’s sense of contribution and significance. Similarly, the mediating effect of organizational psychological ownership highlights another key mechanism through which coaching leadership influences bootlegging innovation behavior. Psychological ownership reflects a deep-seated sense of possession and responsibility toward the organization. When employees develop this sentiment, they become more inclined to act in ways that promote the organization’s long-term well-being and competitiveness (Tanziz & Abadi, 2024). Bootlegging innovation behavior often involves calculated deviations from norms to explore new ideas and can be seen as an expression of such ownership (Augsdorfer, 2022), as employees take proactive steps to address organizational challenges or opportunities without waiting for formal approval. Through an empowering and participatory approach, coaching leadership nurtures this sense of ownership by involving employees in decision-making processes, granting autonomy, and acknowledging individual contributions (O’Donoghue & Van Der Werff, 2022). Therefore, organizations should consider structural and cultural interventions that reinforce feelings of ownership, such as participative goal setting, transparent communication of strategic priorities, and recognition systems that highlight employee-led initiatives. Taken together, these findings suggest that managers seeking to encourage grassroots innovation should focus not only on granting autonomy but also on fostering meaningful work experiences and a sense of psychological ownership among employees.

### 5.4. Limitations and Future Research

Despite the valuable insights offered by this study, we should note several limitations that suggest promising avenues for future research. First, the cross-sectional design of the research constrains the ability to draw robust causal inferences regarding the relationships among coaching leadership, work meaning, organizational psychological ownership, and bootlegging innovation behavior. Although the hypothesized model was developed based on established theoretical foundations and supported by empirical evidence, the possibility of reverse causality or reciprocal relationships cannot be fully ruled out. For instance, employees who frequently engage in bootlegging innovation behavior might perceive higher work meaning or develop a stronger sense of organizational psychological ownership, which, in turn, could influence their perceptions of leadership. Future research should employ longitudinal or experimental designs to strengthen the causal claims and capture the dynamics and evolving nature of these constructs over time.

Second, the specific organizational and cultural contexts in which we collected the data limit the generalizability of these findings. Leadership styles and employee innovation behavior are often shaped by contextual factors such as industry type, organizational climate, and national culture. In particular, the meaning of coaching leadership and the expression of bootlegging behaviors may vary in different sociocultural environments. Future studies could extend the present research by testing the proposed model across diverse cultural backgrounds and organizational settings, thereby enhancing the external validity of the findings. Intercultural comparative research is especially valuable for determining whether the mediating roles of work meaning and organizational psychological ownership are universally applicable or culturally contingent.

Third, while this study highlights work meaning and organizational psychological ownership as sequential mediators in the relationship between coaching leadership and bootlegging innovation behavior, it does not fully address the possibility of additional mediating or moderating mechanisms. Human behavior in organizations is shaped by the complex interplay of individual, team, and organizational factors, and the current model may only partially capture the pathways through which leadership influences bootlegging innovation behavior. Future research could explore alternative or complementary psychological processes, such as psychological empowerment, perceived organizational support, or proactive personality, which might interact with or parallel the roles of work meaning and psychological ownership.

## Figures and Tables

**Table 1:** Confirmatory factor analysis and reliability test.

Variable	Items	Estimate	SE	*p*	AVE	CR	α
Coaching leadership	CL1	0.684	0.028	***	0.474	0.915	0.915
	CL2	0.684	0.028	***			
	CL3	0.662	0.029	***			
	CL4	0.659	0.030	***			
	CL5	0.706	0.027	***			
	CL6	0.701	0.027	***			
	CL7	0.700	0.027	***			
	CL8	0.670	0.029	***			
	CL9	0.725	0.025	***			
	CL10	0.675	0.029	***			
	CL11	0.707	0.026	***			
	CL12	0.688	0.028	***			
Work meaning	WM1	0.757	0.025	***	0.557	0.790	0.790
	WM2	0.730	0.027	***			
	WM3	0.751	0.026	***			
Organizational psychological ownership	OPO1	0.775	0.023	***	0.556	0.834	0.834
	OPO2	0.735	0.026	***			
	OPO3	0.725	0.027	***			
	OPO4	0.748	0.025	***			
Bootlegging innovation behavior	BIB1	0.709	0.029	***	0.572	0.842	0.841
	BIB2	0.754	0.026	***			
	BIB3	0.801	0.024	***			
	BIB4	0.759	0.026	***			
Model fit index	χ^2^(*p*) = 262.398 (0.040), χ^2^/df = 1.171, RMSEA = 0.020, CFI = 0.992, TLI = 0.991, SRMR = 0.029						

Note: *** *p* < 0.001.

**Table 2:** Descriptive and correlation analysis.

Variable	Mean	SD	G	A	EB	T	CL	WM	OPO	BIB
G	1.450	0.498	1							
Age	2.810	1.699	0.040	1						
EB	2.937	1.087	0.105 **	0.085 *	1					
T	2.199	1.511	−0.019	0.881 ***	−0.060	1				
CL	3.688	0.626	−0.026	0.021	0.088 *	0.021	1			
WM	3.808	0.725	0.088 *	0.026	0.112 **	0.048	0.563 ***	1		
OPO	3.748	0.728	0.021	0.049	0.074	0.082 *	0.563 ***	0.669 ***	1	
BIB	3.697	0.751	0.096 **	0.042	0.065	0.048	0.467 ***	0.466 ***	0.459 ***	1

Note: *** *p* < 0.001, ** *p* < 0.01, * *p* < 0.05. G: gender; A: age, EB: educational background; T: tenure; CL: coaching leadership; WM: work meaning; OPO: organizational psychological ownership; BIB: bootlegging innovation behavior.

**Table 3:** Standardized path analysis.

Path	Std. Estimate	S.E.	t	*p*	LLCI	ULCI
Coaching leadership → Work meaning	0.530	0.040	13.193	0.000	0.447	0.603
Coaching leadership → Organizational psychological ownership	0.193	0.042	4.624	0.000	0.109	0.275
Coaching leadership → Bootlegging innovation behavior	0.217	0.057	3.779	0.000	0.105	0.330
Work meaning → Organizational psychological ownership	0.633	0.038	16.749	0.000	0.553	0.703
Work meaning → Bootlegging innovation behavior	0.158	0.065	2.444	0.015	0.033	0.285
Organizational psychological ownership → Bootlegging innovation behavior	0.204	0.065	3.166	0.002	0.078	0.334

**Table 4:** Standardized bootstrap indirect effect test.

Indirect Effect	Std. Effect	Boot SE	Boot LLCI	Boot ULCI
Coaching leadership → Work meaning → Bootlegging innovation behavior	0.084	0.035	0.016	0.155
Coaching leadership → Organizational psychological ownership → Bootlegging innovation behavior	0.040	0.015	0.015	0.077
Coaching leadership → Work meaning → Organizational psychological ownership → Bootlegging innovation behavior	0.069	0.023	0.027	0.118
Total	0.192	0.033	0.132	0.259

Note: N = 427. The indirect effect was tested for significance using 95% bias-corrected bootstrapped confidence intervals. Bootstrap resampling = 5000.

**Table 5:** Unstandardized path analysis.

Path	Estimate	S.E.	t	*p*	LLCI	ULCI
Coaching leadership → Work meaning	0.569	0.045	12.529	0.000	0.480	0.656
Coaching leadership → Organizational psychological ownership	0.208	0.045	4.602	0.000	0.117	0.295
Coaching leadership → Bootlegging innovation behavior	0.235	0.062	3.823	0.000	0.114	0.356
Work meaning → Organizational psychological ownership	0.633	0.043	14.688	0.000	0.547	0.715
Work meaning → Bootlegging innovation behavior	0.160	0.065	2.468	0.014	0.033	0.284
Organizational psychological ownership → Bootlegging innovation behavior	0.207	0.066	3.113	0.002	0.075	0.338

**Table 6:** Unstandardized bootstrap indirect effect test.

Indirect Effect	Effect	Boot SE	Boot LLCI	Boot ULCI
Coaching leadership → Work meaning → Bootlegging innovation behavior	0.091	0.038	0.019	0.168
Coaching leadership → Organizational psychological ownership → Bootlegging innovation behavior	0.043	0.017	0.016	0.083
Coaching leadership → Work meaning → Organizational psychological ownership → Bootlegging innovation behavior	0.075	0.025	0.029	0.130
Total	0.444	0.057	0.328	0.550

Note: N = 427. The indirect effect was tested for significance using 95% bias-corrected bootstrapped confidence intervals. Bootstrap resampling = 5000.

**Table 7:** Robustness Check of Mediator Ordering.

Effect	Proposed Model β	95% CI	Reverse Model β	95% CI
CL → WM → BIB	0.091	[0.019, 0.168]	0.034	[0.007, 0.069]
CL → OPO → BIB	0.043	[0.016, 0.083]	0.117	[0.046, 0.201]
Serial indirect effect	0.075	[0.029, 0.130]	0.057	[0.013, 0.108]
Total indirect effect	0.209	[0.144, 0.282]	0.209	[0.144, 0.282]
Direct effect	0.235	[0.114, 0.356]	0.235	[0.114, 0.356]
Total effect	0.444	[0.328, 0.550]	0.444	[0.328, 0.550]

Note. CL = coaching leadership; WM = work meaning; OPO = organizational psychological ownership; BIB = bootlegging innovation behavior. The proposed model specifies the sequence of coaching leadership → work meaning → organizational psychological ownership → bootlegging innovation behavior, whereas the reverse-order model specifies coaching leadership → organizational psychological ownership → work meaning → bootlegging innovation behavior. Both models were saturated (df = 0); therefore, global fit indices were identical and are not informative for substantive model comparison. Accordingly, the robustness check focused on indirect effects and explained variance. 95% CI = bias-corrected bootstrap confidence interval based on 5000 bootstrap samples.

## Data Availability

The data supporting this study’s findings are available from the corresponding author upon reasonable request.

## References

[B1-behavsci-16-00484] Agarwal U. A., Datta S., Blake-Beard S., Bhargava S. (2012). Linking LMX, innovative work behaviour and turnover intentions: The mediating role of work engagement. Career Development International.

[B2-behavsci-16-00484] Almrshed S. K. H., Jasim H. M., Hassan A. S. (2023). The effect of innovation management on sustainable competitive advantage in contemporary organizations. Journal of Law and Sustainable Development.

[B3-behavsci-16-00484] Alshahrani M. A., Yaqub M. Z., Ali M., El Hakimi I., Salam M. A. (2025). Could entrepreneurial leadership promote employees’ IWB? The roles of intrinsic motivation, creative self-efficacy and firms’ innovation climate. International Journal of Innovation Science.

[B4-behavsci-16-00484] Anderson V. (2013). A Trojan horse? The implications of managerial coaching for leadership theory. Human Resource Development International.

[B5-behavsci-16-00484] Artis A. B., Harris E. G. (2007). Self-directed learning and sales force performance: An integrated framework. Journal of Personal Selling & Sales Management.

[B6-behavsci-16-00484] Augsdorfer P. (2022). Corporate underground: Bootleg innovation and constructive deviance.

[B7-behavsci-16-00484] Bagozzi R. P., Yi Y. (1988). On the evaluation of structural equation models. Journal of the Academy of Marketing Science.

[B8-behavsci-16-00484] Bai T., Jia D., Liu S., Shahzad F. (2024). Psychological ownership and ambidexterity influence the innovative work behavior and job performance of SME employees: A mediating role of job embeddedness. Current Psychology.

[B9-behavsci-16-00484] Bailey C., Madden A. (2017). Time reclaimed: Temporality and the experience of meaningful work. Work, Employment and Society.

[B10-behavsci-16-00484] Blau P. M. (1964). Exchange and power in social life.

[B11-behavsci-16-00484] Brown G., Pierce J. L., Crossley C. (2014). Toward an understanding of the development of ownership feelings. Journal of Organizational Behavior.

[B12-behavsci-16-00484] Cardon P. (2019). Communication on internal digital platforms. Exploring internal communication.

[B13-behavsci-16-00484] Cascio W., Boudreau J. (2010). Investing in people: Financial impact of human resource initiatives.

[B14-behavsci-16-00484] Chai D. S., Song J. H., You Y. M. (2020). Psychological ownership and openness to change: The mediating effects of work engagement, and knowledge creation. Performance Improvement Quarterly.

[B15-behavsci-16-00484] Cheney G., Zorn T. E., Planalp S., Lair D. J. (2008). Meaningful work and personal/social well-being organizational communication engages the meanings of work. Annals of the International Communication Association.

[B16-behavsci-16-00484] Criscuolo P., Salter A., Ter Wal A. L. (2014). Going underground: Bootlegging and individual innovative performance. Organization Science.

[B17-behavsci-16-00484] Cui Z., Wang H., Nanyangwe C. N. (2022). How does coaching leadership promote employee’s constructive deviance? Affective events perspective. Leadership & Organization Development Journal.

[B18-behavsci-16-00484] Dawkins S., Tian A. W., Newman A., Martin A. (2017). Psychological ownership: A review and research agenda. Journal of Organizational Behavior.

[B19-behavsci-16-00484] Deci E. L., Ryan R. M. (2000). The “what” and “why” of goal pursuits: Human needs and the self-determination of behavior. Psychological Inquiry.

[B20-behavsci-16-00484] Edmondson A. C. (2018). The fearless organization: Creating psychological safety in the workplace for learning, innovation, and growth.

[B21-behavsci-16-00484] Gardner W. L., Schermerhorn J. R. (2004). Unleashing individual potential: Performance gains through positive organizational behavior and authentic leadership. Organizational Dynamics.

[B22-behavsci-16-00484] Gilliam T. H. (2024). The principal’s coach: A phenomenological exploration of psychological safety and coaching dynamics in educational leadership. Doctoral dissertation.

[B23-behavsci-16-00484] Glavas A., Kelley K. (2014). The effects of perceived corporate social responsibility on employee attitudes. Business Ethics Quarterly.

[B24-behavsci-16-00484] Globocnik D. (2023). Individual and contextual factors affecting employees’ inclination to bootlegging. Corporate underground: Bootleg innovation and constructive deviance.

[B25-behavsci-16-00484] Globocnik D., Peña Häufler B., Salomo S. (2022). Organizational antecedents to bootlegging and consequences for the newness of the innovation portfolio. Journal of Product Innovation Management.

[B26-behavsci-16-00484] Globocnik D., Salomo S. (2015). Do formal management practices impact the emergence of bootlegging behavior?. Journal of Product Innovation Management.

[B27-behavsci-16-00484] Goenaga R. R. (2024). Employee perceptions of effective leadership styles in promoting employee motivation in a governmental academic workplace. Doctoral dissertation.

[B28-behavsci-16-00484] Göçen A. (2021). How do teachers perceive meaningful leadership? Overview of a qualitative exploration. Journal of Pedagogical Research.

[B29-behavsci-16-00484] Guarana C. L., Avolio B. J. (2022). Unpacking psychological ownership: How transactional and transformational leaders motivate ownership. Journal of Leadership & Organizational Studies.

[B30-behavsci-16-00484] Hair J. F., Matthews L. M., Matthews R. L., Sarstedt M. (2017). PLS-SEM or CB-SEM: Updated guidelines on which method to use. International Journal of Multivariate Data Analysis.

[B31-behavsci-16-00484] Hao J. X., Chen Z., Mahsud M., Yu Y. (2024). Organizational psychological ownership and innovative work behavior: The roles of coexisting knowledge sharing and hiding across organizational contexts. Journal of Knowledge Management.

[B32-behavsci-16-00484] Hoßbach C., Bachmann M., Roth K., Neyer A. K. (2024). Moving towards a data-driven approach to self-leadership: Exploring the combination of app-based reflection diaries and data-based ideation techniques. International Journal of Entrepreneurship and Innovation Management.

[B33-behavsci-16-00484] Huang D., Zhu T., Wu Y., Sun T. (2022). A study on paradoxical leadership and multiple path mechanisms of employees’ bootleg innovation. Psychology Research and Behavior Management.

[B34-behavsci-16-00484] Iqbal S., Bureš V., Zanker M., Abdullah M., Tootell B. (2023). A system dynamics perspective on workplace spirituality and employee behavior. Administrative Sciences.

[B35-behavsci-16-00484] Jia J., Liu Z., Liu W., Hu J. (2023). Promotion mechanism of high-involvement human resource management practices to employees’ bootlegging: A moderated mediation model. Frontiers in Psychology.

[B36-behavsci-16-00484] Jong J. P. J. (2007). Individual innovation: The connection between leadership and employees’ innovative work behavior.

[B37-behavsci-16-00484] Kim M., Beehr T. A. (2017). Self-efficacy and psychological ownership mediate the effects of empowering leadership on both good and bad employee behaviors. Journal of Leadership & Organizational Studies.

[B38-behavsci-16-00484] Lee M. C. C., Idris M. A., Tuckey M. (2019). Supervisory coaching and performance feedback as mediators of the relationships between leadership styles, work engagement, and turnover intention. Human Resource Development International.

[B39-behavsci-16-00484] Liu F., Chow I. H. S., Zhang J. C., Huang M. (2019). Organizational innovation climate and individual innovative behavior: Exploring the moderating effects of psychological ownership and psychological empowerment. Review of Managerial Science.

[B40-behavsci-16-00484] Lysova E. I., Fletcher L., El Baroudi S. (2023). What enables us to better experience our work as meaningful? The importance of awareness and the social context. Human Relations.

[B41-behavsci-16-00484] Malik P., Garg P. (2020). Learning organization and work engagement: The mediating role of employee resilience. The International Journal of Human Resource Management.

[B42-behavsci-16-00484] Masood A., Zhang Q., Singh N., Meena B., Perano M. (2024). Exploring leader’s unethical proorganizational behavior and follower attitudes toward knowledge hiding and sharing in the service industry: A social learning perspective. Journal of Knowledge Management.

[B43-behavsci-16-00484] Masterson S. S., Stamper C. L. (2003). Perceived organizational membership: An aggregate framework representing the employee–organization relationship. Journal of Organizational Behavior: The International Journal of Industrial, Occupational and Organizational Psychology and Behavior.

[B44-behavsci-16-00484] Matei R., Veith C. (2023). Empowerment and engagement: The role of autonomy and feedback in fostering employee motivation. Manager.

[B45-behavsci-16-00484] McShane L., Cunningham P. (2012). To thine own self be true? Employees’ judgments of the authenticity of their organization’s corporate social responsibility program. Journal of Business Ethics.

[B46-behavsci-16-00484] Milner J., McCarthy G., Milner T. (2018). Training for the coaching leader: How organizations can support managers. Journal of Management Development.

[B47-behavsci-16-00484] Nanyangwe C. N., Wang H., Cui Z. (2021). Work and innovations: The impact of self-identification on employee bootlegging behaviour. Creativity and Innovation Management.

[B48-behavsci-16-00484] Neves R. (2024). The engineering leadership playbook: Strategies for team success and business growth.

[B49-behavsci-16-00484] O’Donoghue D., Van Der Werff L. (2022). Empowering leadership: Balancing self-determination and accountability for motivation. Personnel Review.

[B50-behavsci-16-00484] Owens B. P., Hekman D. R. (2012). Modeling how to grow: An inductive examination of humble leader behaviors, contingencies, and outcomes. Academy of Management Journal.

[B51-behavsci-16-00484] Pierce J. L., Kostova T., Dirks K. T. (2001). Toward a theory of psychological ownership in organizations. Academy of Management Review.

[B52-behavsci-16-00484] Rana S., Ardichvili A., Polesello D. (2016). Promoting self-directed learning in a learning organization: Tools and practices. European Journal of Training and Development.

[B53-behavsci-16-00484] Robinson Y. (2024). Analyzing the impact of coaching leadership style on employee engagement.

[B54-behavsci-16-00484] Salleh W., Rezal W. S. (2021). The study of bootlegging initiatives in service organization. Doctoral dissertation.

[B55-behavsci-16-00484] Sen A. (1993). Internal consistency of choice. Econometrica: Journal of the Econometric Society.

[B56-behavsci-16-00484] Spreitzer G. M. (1995). Psychological empowerment in the workplace: Dimensions, measurement, and validation. Academy of Management Journal.

[B57-behavsci-16-00484] Steger M. F. (2016). Creating meaning and purpose at work. The Wiley Blackwell handbook of the psychology of positivity and strengths-based approaches at work.

[B58-behavsci-16-00484] Steger M. F., Dik B. J., Duffy R. D. (2012). Measuring meaningful work: The work and meaning inventory (WAMI). Journal of Career Assessment.

[B59-behavsci-16-00484] Surma-aho A., Kirjavainen S., Björklund T. A. (2024). It ain’t over till it’s over: Adjusting the intensity and conformity of championing efforts after initial failure. Creativity and Innovation Management.

[B60-behavsci-16-00484] Tanziz M. T., Abadi F. (2024). The role of psychological ownership and organizational justice regarding knowledge-sharing behavior with perception of organizational support as moderation role (case study in United Tractors Group). 5th International Conference on Global Innovation and Trends in Economy 2024 (INCOGITE 2024).

[B61-behavsci-16-00484] Toni M., Mehta A. K., Chandel P. S., MK K., Selvakumar P. (2025). Mentoring and coaching in staff development. Innovative approaches to staff development in transnational higher education.

[B62-behavsci-16-00484] Torres I. C. O. (2024). Coaching to leadership trust towards an intrapreneurial framework. International Multidisciplinary Research Journal.

[B63-behavsci-16-00484] Valtonen A., Kimpimäki J. P., Malacina I. (2023). From ideas to innovations: The role of individuals in idea implementation. Creativity and Innovation Management.

[B64-behavsci-16-00484] Van Dyne L., Pierce J. L. (2004). Psychological ownership and feelings of possession: Three field studies predicting employee attitudes and organizational citizenship behavior. Journal of Organizational Behavior: The International Journal of Industrial, Occupational and Organizational Psychology and Behavior.

[B65-behavsci-16-00484] Walumbwa F. O., Hartnell C. A., Oke A. (2010). Servant leadership, procedural justice climate, service climate, employee attitudes, and organizational citizenship behavior: A cross-level investigation. Journal of Applied Psychology.

[B66-behavsci-16-00484] Wang X. L., Wang M. Y., Liu J. N. (2023). Study on the influence mechanism of leaders’ abusive supervision on employees’ bootlegging innovation behavior. International Journal of Conflict Management.

[B67-behavsci-16-00484] Wefald A. J. (2024). Navigating the coaching and leadership landscape: Strategies and insights for success: Strategies and insights for success.

[B68-behavsci-16-00484] Weick K. E., Sutcliffe K. M. (2011). Managing the unexpected: Resilient performance in an age of uncertainty.

[B69-behavsci-16-00484] Yuan F., Woodman R. W. (2010). Innovative behavior in the workplace: The role of performance and image outcome expectations. Academy of Management.

[B70-behavsci-16-00484] Zhang J., Choi M., Wang K., Kim H. E. (2025). How can ethical leadership increase employees’ bootlegging innovation behavior in China?: A serial mediation model of psychological wellbeing and psychological entitlement. Frontiers in Psychology.

[B71-behavsci-16-00484] Zhang L., Qin G., Yang F., Jiang P. (2023). Linking leader humor to employee bootlegging: A resource-based perspective. Journal of Business and Psychology.

[B72-behavsci-16-00484] Zhang X. (2020). The relationship of coaching leadership and innovation behavior: Dual mediation model for individuals and teams across levels. Journal of Leadership.

[B73-behavsci-16-00484] Zhang Y., Liu G., Zhang L., Xu S., Cheung M. W. L. (2021). Psychological ownership: A meta-analysis and comparison of multiple forms of attachment in the workplace. Journal of Management.

[B74-behavsci-16-00484] Zighan S., Ruel S. (2023). SMEs’ resilience from continuous improvement lenses. Journal of Entrepreneurship in Emerging Economies.

